# Rapid automatic naming and phonological awareness deficits in preschool children with probable developmental coordination disorder

**DOI:** 10.3389/fped.2022.957823

**Published:** 2022-07-27

**Authors:** Hsiang-Chun Cheng, Rong-Ju Cherng, Pei-Yu Yang

**Affiliations:** ^1^Department of Speech Language Pathology and Audiology, Hungkuang University, Taichung, Taiwan; ^2^Department of Physical Therapy, Hungkuang University, Taichung, Taiwan; ^3^Department of Physical Therapy, College of Medicine, National Cheng Kung University, Tainan, Taiwan; ^4^Institute of Allied Health Sciences, College of Medicine, National Cheng Kung University, Tainan, Taiwan; ^5^Department of Physical Medicine and Rehabilitation, China Medical University Hospital, Taichung, Taiwan; ^6^School of Medicine, China Medical University, Taichung, Taiwan

**Keywords:** developmental coordination disorder, rapid automatic naming, phonological awareness, visual perceptual-motor coordination, manual dexterity

## Abstract

Children with developmental coordination disorder (DCD) have been reported to have a higher risk of dyslexia than children with typical development (TD). Phonological awareness (PA) and rapid automatic naming (RAN) are known to be predictive of children’s reading development. The present study examined PA and RAN in preschool children with and without probable DCD in Taiwan. In total, 704 children aged 5–6 years old from 25 preschools in Taichung City were included as participants. The probable DCD children performed more poorly than the children with TD on the PA and the RAN tests. Put in deficit terms, 22% of the children with TD, but 48% of the probable DCD children, had a single or dual PA/RAN deficit. Furthermore, it was manual dexterity that bore the unique relationship with RAN. Automatic visual perceptual-motor coordination may be the common processing component that underlies RAN and probable DCD. The early visual perceptual-motor profile of probable DCD children has not been well recognized before.

## Introduction

Children with developmental coordination disorder (DCD) are characterized by motor skill difficulties that significantly interfere with academic achievement and activities of daily living, these problems are not better explained by intellectual delay, visual impairment, or other neurological conditions that affect movement (1). The prevalence rate of DCD in children aged 5–11 years old was estimated to be 6% by the American Psychological Association (2). However, prevalence varies across countries, ranging from 1.8 to 19% (3), and was reported to be 12.4% in children aged 5–6 in Taiwan (4).

The etiology and prognosis of DCD are still poorly understood. Researchers and practitioners have now generally agreed that DCD is not a uniform disorder, and an awareness of the existence of subtypes and comorbidities is necessary (5). For example, studies have found that children with DCD are not just impaired in sensorimotor skills but also have difficulties in non-motor tasks such as attention deficits (6, 7), visuospatial working memory deficits (8, 9), specific language impairments (4, 10), learning disabilities (11, 12), oral-motor function (13), and cognitive deficits (14–17). Children with DCD have also been reported to have difficulties in performing dual-motor tasks (18, 19).

Among the diverse comorbid conditions associated with DCD, language impairments call for special attention for both theoretical and practical reasons. Theoretically, motor control and related perceptual-motor coordination are clearly implicated in language processing, reading in particular. A common mechanism may underlie the impairments of both motor control and language control. Investigations of the nature of the comorbidity of motor deficits and language impairments could shed light on this common mechanism. Practically speaking, language abilities, particularly reading, are crucial for children’s learning in school and their later academic achievements. Recognition of DCD children’s difficulties in processing language allows for early interventions and remediation. The practical reason has, in fact, led many researchers to study children with language impairments (e.g., specific language impairment and dyslexia) when they investigated the nature of motor-language comorbidity (4, 20). Only a few studies targeted DCD in their investigations (4, 10, 20, 21). Archibald and Alloway (10) noted that “Some children with DCD may have a less common language profile which may go unrecognized” and stressed that “further examination of the language needs of children with DCD is warranted.” The purpose of the present study, therefore, was to examine the performances of DCD children in two important tasks known to be related to reading ability [rapid automatized naming (RAN) and phonological awareness (PA)] and to learn how they fared with the typically developing children.

Phonological awareness (PA) is a metalinguistic ability that captures the composability and sound structure of speech. Continuous speech is made up of discrete sounds called phonemes and phonemes are organized in specific ways to form syllables, words, and sentences. The ability allows speakers to manipulate the phonemes in a word, i.e., to pronounce a word when a phoneme is added, deleted, or replaced. Because reading involves mapping orthography to phonology or translating graphemes to phonemes, PA has been found to play a critical role in reading development, especially among beginners and poor readers (22). This is true even in Chinese, where the writing system does not have a transparent orthography-to-phonology mapping. A different kind of PA (of onsets and rhymes, but not phonemic awareness) was found to predict reading success in Chinese (23).

Naming speed has also been found to predict reading ability, particularly reading fluency. A task commonly used to assess naming speed is RAN (24). In its original design, the task measures the speed with which a child can continuously name an array of 50 stimuli in one of four symbolic categories: letters, numbers, colors, and objects. Numerous studies have established that RAN is one of the best, perhaps universal, predictors of reading fluency across all known orthographies (25, 26). Wolf et al. proposed that the naming speed deficit was independent of the PA deficit and constituted the second deficit in developmental dyslexia (27). With respect to Chinese, RAN was shown to be a better predictor than PA of reading difficulties in Chinese-speaking children (28).

In light of the important roles of PA and naming speed in predicting children’s reading development, we set out to investigate these two abilities in children with DCD and examine how they fared with typically developing children. The findings of the investigation would be useful for designing early intervention programs to address the linguistic needs of children with DCD.

## Materials and methods

### Participants and procedure

A total of 744 children (boys: 416 and girls: 328) aged 5–6 years old from 25 preschools in Taichung City, Taiwan, passed the screening examination, which ruled out neurological, musculoskeletal, and cardiopulmonary system impairments. The parents of the participating children provided informed consent before the testing sessions. The study protocol was approved by the Institutional Review Board of Kuang Tien General Hospital No. 1012.

### Tests and measures

#### Test of non-verbal intelligence—Chinese version

Test of Non-verbal Intelligence—Chinese version (C-TONI, 3rd ed.) (29) is a test for non-verbal intelligence, which was originally developed by Brown et al. (30). It has been revised and translated into many languages and is widely used in non-English-speaking countries. The Chinese version (C-TONI-3) was translated from the third version of TONI and established by Wu et al. (29). The C-TONI-3 was standardized for children between the ages of 4–16 years and 5 months. It contains 45 test items and provides the norm-referenced scores of a percentile rank and IQ scores. The cutoff score in the manual’s suggestion used for the C-TONI-3 is at or below the 70 standard scores of the age-related norm. It is widely used in Taiwan as a screening test for non-verbal IQ due to its high test-retest reliability (*r* = 0.84–0.91), objectivity, and quick administration.

### Peabody picture vocabulary test-revised—Chinese version

The peabody picture vocabulary test-revised-Chinese version (C-PPVT-R) was used to determine the vocabulary comprehension of the children. PPVT was originally developed by Dunn (31) and was revised by Dunn and Dunn in 1981 (PPVT-R) (32, 33). The test has been widely used as a standard measure of receptive vocabulary and a screening test of verbal ability. The Chinese version of C-PPVT-R was based on a revision of PPVT-R and was tested on 886 children between the ages of 3 and 12 years (33). Due to its objectivity, rapid scoring, quick administration time (10–15 min), high split-half reliability (*r* = 0.90–0.97), and high test-retest reliability (*r* = 0.84–0.90), we chose this test as a measure of vocabulary comprehension. The C-PPVT-R is norm-referenced, and a cutoff score is established as the 10th percentile of the age-related norm, with a deficit being determined if the test score is at or below the cutoff.

### Movement sssessment battery for children-second edition (movement ABC-2)

Movement Assessment Battery for Children-Second Edition (MABC-2) was used to test the motor ability of the children in this study (34). This is a standardized test that consists of two parts: a checklist for the teacher or parent to assess a child’s behavior, and a formal test composed of eight items categorized into three subtests of the manual, namely, dexterity, aiming and catching, and balance. Scores for each component can be converted to standard scores, and percentile ranks can be calculated from the components. According to the test manual, children scoring at or below the 5th percentile of the total score can be diagnosed with high-risk DCD; a score between the 5th and the 16th percentiles is indicative of mild-risk DCD; probable DCD (≤15th percentile), and typical development (TD; >15th percentile). Due to its high reliability and validity, the MABC-2 has been widely applied in research and clinics as a diagnostic tool for children with DCD (35, 36). In the current study, we adopted below the 16th percentile as the cutoff for diagnosing probable DCD.

### Phonological awareness test—Chinese version

Phonological awareness test—Chinese version (37) is a standardized and norm-referenced test that includes 24 items categorized into three subtests of onset detection, rhyme detection, and tone detection. Each test item provides three choices, only one of which is correct, and one point is given for the correct answer. A higher score thus indicates a better performance. The score for each item can be summed to get a subtest score, and the three subtest scores can then be summed to get a total score, which is then converted to a percentile rank score. A cutoff score is established at the 25th percentile of the age-related norm for the total score of the PA.

### Rapid automatized naming test (RAN test)

The RAN test measures how quickly individuals can accurately name objects, colors, or symbols (letters or digits) (38). The stimuli for the task used in this study for preschoolers included five digits (1, 2, 3, 4, and 5), five colors (red, yellow, blue, white, and black), and five objects (hand, door, bowl, tree, and pig), constituting three subtests. The stimuli were presented on standardized 200 mm × 130 mm size cards and were arranged in 10 × 5 randomly ordered arrays.

The child was asked to look at the cards and read the stimuli as quickly as possible, moving from left to right and top to bottom. The score was based on the naming time. The naming time was the time interval (measured in seconds) between the response to the first stimulus and the response to the last stimulus. If an error or an omission occurred, the naming time would be penalized with an extra time which was 0.25 s for each error or omission. The lower the score, the better the performance. A cutoff score is established at the 10th percentile of the age-related norm. Previous studies showed that letters can only be validly evaluated after children have entered elementary school, whereas objects, digits, and colors can be tested at an earlier stage (39, 40). We thus used objects, digits, and colors in our RAN tests.

### Procedure

Parents completed a questionnaire for ruling out attention-deficit hyperactivity disorder. All the children were then individually assessed with C-PPVT-R, C-TONI-3, and MABC-2. In total, 40 children who scored below the IQ screening tests (C-PPVT-R or C-TONI-3) were excluded from the study. Based on the results of the motor test, 704 children were classified into one of two groups: children with probable DCD (64) and typical development (TD) children (640). They have then given the PA and the RAN tests.

### Data analysis

The independent-sample *t*-test was used to compare the age and the scores of the probable DCD and the TD children on the C-TONI-3, C-PPVT-R, and MABC-2 tests. Contingency tables with chi-square analyses were performed to compare the gender distributions of the two groups of children, and for the examination of comorbidity. The analysis of covariance (ANCOVA) was applied to compare the PA and RAN scores between the two groups of children with C-TONI-3 and C-PPVT-R were controlled. Partial correlation analyses were applied to examine the relationships among the scores on PA, RAN, and MABC-2 after C-TONI-3 and C-PPVT-R were controlled. The multiple linear regression analysis was used to predict the strength of motor ability for PA and RAN abilities, and to determine the association between the subtypes of PA (onset, rhyme, and tone) and the subtypes of MABC-2 (manual dexterity, aiming and catching, and balance), and the relationship between three subtypes of RAN (digits RAN, color RAN, and object RAN) and the subtypes of MABC-2. The significant level for all of the tests was set at 0.05.

Analysis of covariance (ANCOVA) was applied to compare the scores of the two groups of children on PA and RAN with C-TONI-3 and C-PPVT-R were controlled.

## Results

### Prevalence of developmental coordination disorder

Among the 704 children, 64 (9%) were diagnosed with probable DCD according to their scores on the MABC test. Boys and girls had similar prevalence rates (9.18 and 8.97%, respectively).

### Comparisons of developmental coordination disorder and typical development on key characteristics

Table 1 summarizes the characteristics of the children with probable DCD and TD. There were no significant differences in the distributions of age and gender between the two groups of children. However, the probable DCD children showed significantly lower C-TONI-3 and C-PPVT-R scores than the TD children. Nonetheless, the scores all fell within the normal range and thus the probable DCD children were not considered as having an intellectual deficiency. The probable DCD children also had significantly lower scores in all subtests of MABC-2 than the TD children.

**TABLE 1:** The results of the statistical tests that compared key characteristics of the DCD children and the TD children.

Key characteristics	DCD (*N* = 64)	TD (*N* = 640)	*P*-value
Age	6.08 ± 0.45	6.09 ± 0.34	0.94
Gender (M:F)	36:28	356:284	0.92
C-PPVT-R (standard score)	105.03 ± 11.10	110.22 ± 12.10	0.001
C-TONI-3 (standard score)	89.30 ± 13.37	96.05 ± 16.08	0.0000
**MABC-2**			
Manual dexterity (standard score)	7.08 ± 2.25	12.00 ± 2.64	0.0000
Aiming and catching (standard score)	5.45 ± 2.34	9.56 ± 2.94	0.0000
Balance (standard score)	8.20 ± 2.55	12.10 ± 2.52	0.0000
Total score (standard score)	6.09 ± 1.09	11.59 ± 2.32	0.0000
Total score (percentile rank)	10.53 ± 5.17	65.62 ± 22.43	0.0000

Shown in the table are the mean and the standard deviation for each characteristic, except for gender, where numbers of males and females are given.

### Comparisons of developmental coordination disorder and typical development on phonological awareness and rapid automatic naming

The performance scores on the PA test and the RAN test for the probable DCD children and the TD children are presented in Table 2. The probable DCD children had significantly lower scores (i.e., lower performance) than the TD children on the overall PA test and on each of the subtests. The sizes of the differences suggest that PA of the onset of a syllable appeared to be more difficult than that of the rhyme or the tone for the probable DCD children relative to the TD children. The probable DCD also had significantly slower naming responses (i.e., longer naming times) than the TD children on all three RAN subtests.

**TABLE 2:** Means and standard deviations of the scores on the phonological awareness test and the rapid automatized naming test for DCD children and TD children.

Test	DCD	TD	*P*-value
Phonological awareness (in scores)	12.30 ± 4.73	14.55 ± 4.65	0.0000
Onset	3.94 ± 1.83	4.97 ± 1.96	0.004
Rhyme	4.52 ± 1.77	5.10 ± 1.95	0.21
Tone	3.84 ± 2.36	4.47 ± 2.17	0.21
Rapid automatized naming (in seconds)	65.91 ± 18.18	55.29 ± 13.83	0.0001
Digit naming	45.62 ± 16.06	37.08 ± 11.74	0.000
Color naming	78.23 ± 33.99	65.14 ± 20.70	0.000
Object naming	73.89 ± 18.25	63.73 ± 15.86	0.000

P-values are the results of the statistical tests comparing the two groups of children after the C-TONI-3 and C-PPVT-R scores were controlled.

### Correlations of phonological awareness and rapid automatic naming with MABC-2

Table 3 gives the Pearson’s partial correlation coefficients between the children’s scores on the MABC-2 and their scores on the PA test and the RAN test, with their C-TONI-3 and C-PPVT-R scores partially out. There were significant correlations between the children’s PA performances and their motor performances. Judged from the sizes of the correlations and their statistical significance, manual dexterity appeared to be the most relevant aspect of motor control that was related to PA. There were also significant correlations between the children’s RAN performances and their motor performances. Again, manual dexterity appeared to be the more relevant aspect of motor control that was related to RAN.

**TABLE 3:** Pearson partial correlation coefficients relating the children’s scores on the phonological awareness test and their scores on the rapid automatized naming test to their scores on the motor test, after the C-TONI-3 and C-PPVT-R scores were controlled.

	1	2	3	4	5	6
(1) Phonological awareness	1					
(2) Rapid automatized naming	−0.27**	1				
(3) Total score of MABC-2	0.13**	−0.25**	1			
(4) Manual dexterity	0.14**	−0.25**	0.72**	1		
(5) Aiming and catching	0.07	−0.19**	0.72**	0.27**	1	
(6) Balance	0.07	−0.08*	0.57**	0.20**	0.25**	1

*p < 0.05, **p < 0.01.

There was a significant correlation between the children’s performances on the PA test and their performances on the RAN test: *r* = −0.27 (*p* < 0.0001), even when their C-TONI-3 and C-PPVT-R scores were controlled (Table 3). This means that the children who had better PA also named digits, colors, and objects faster. Because PA and RAN were correlated, we repeated the partial correlational analysis of PA and MABC-2 by controlling for C-TONI-3, C-PPVT-R, and RAN (Table 4). The partial correlation between the PA total scores and the MABC-2 total scores was 0.06 and not significant (*p* = 0.09). We also ran the partial correlational analysis of RAN and MABC-2 by controlling for C-TONI-3, C-PPVT-R, and PA. The partial correlation between the RAN average scores and the MABC-2 total scores was −0.23 and significant (*p* < 0.0001) (Table 4).

**TABLE 4:** The partial correlational analysis of PA/RAN and MABC-2 by controlling for C-TONI-3, C-PPVT-R, and RAN/PA.

Control variables		PA/RAN	Total score of MABC-2
C-TONI-3, C-PPVT-R scores, and RAN were controlled	PA	1.00	0.06
C-TONI-3, C-PPVT-R scores, and RAN were controlled	RAN	1.00	−0.23**

**p < 0.01.

### Relationship between the subtest of MABC-2 and the onset of phonological awareness, and the subtest of rapid automatic naming in children with probable developmental coordination disorder and typical development

The subtest of MABC-2 for manual dexterity, aiming and catching, and balance function was used as independent variables; onset of PA and subtests of the RAN (digits RAN, color RAN, and object RAN) as the dependent variables, and then the C-PPVT-R and C-TONI-3 as the control variables in the multiple linear regression. The results are presented in Tables 5–8.

**TABLE 5:** Linear regression analysis for the predictive effect of subtest of MABC-2 on the onset of PA.

Predictor	*R*	*R* ^2^	Δ*R*^2^	*F*	Beta	*t*	Sig.
C-TONI-3	0.33	0.11	0.11	86.23	0.26	6.81	0.00
C-PPVT-R	0.36	0.13	0.02	52.04	0.14	3.75	0.00
Manual dexterity	0.37	0.14	0.01	38.26	0.09	2.51	0.01
Aiming and catching	–	–	–	–	–	–	–
Balance	0.39	0.15	0.01	30.38	0.09	2.44	0.01

**TABLE 6:** Linear regression analysis for the predictive effect of subtest of MABC2 on digits RAN.

Predictor	*R*	*R* ^2^	Δ*R*^2^	*F*	Beta	*t*	Sig.
C-PPVT-R	–	–	–	–	–	–	–
C-TONI-3	0.31	0.09	0.09	86.23	0.26	6.81	0.00
Manual dexterity	0.38	0.14	0.05	38.26	0.09	2.51	0.01
Aiming and catching	0.38	0.15	0.01	30.38	0.09	2.44	0.01
Balance	–	–	–	–	–	–	–

**TABLE 7:** Linear regression analysis for the predictive effect of subtest of MABC2 on color RAN.

Predictor	*R*	*R* ^2^	Δ*R*^2^	*F*	Beta	*t*	Sig.
C-PPVT-R	–	–	–	–	–	–	–
Manual dexterity	0.20	0.04	0.04	28.04	−0.14	−3.54	0.00
C-TONI-3	0.24	0.06	0.02	21.69	−0.14	−3.67	0.00
Aiming and catching	0.27	0.07	0.01	18.51	−0.13	−3.39	0.00
Balance	–	–	–	–	–	–	–

**TABLE 8:** Linear regression analysis for the predictive effect of subtest of MABC-2 on object RAN.

Predictor	*R*	*R* ^2^	Δ*R*^2^	*F*	Beta	*t*	Sig.
Manual dexterity	0.29	0.09	0.09	65.58	−0.23	−6.21	0.00
C-TONI-3	0.35	0.12	0.04	49.81	−0.20	−5.45	0.00
Aiming and catching	0.37	0.13	0.03	35.96	−0.10	−2.71	0.00
C-PPVT-R	–	–	–	–	–	–	–
Balance	–	–	–	–	–	–	–

Table 5 showed that aiming and catching is not a predictor for onset of the PA. When controlling C-PPVT-R, and C-TONI-3, the manual dexterity and balance of the children have a significant predictive effect on the onset of the PA (β = 0.09, *p* < 0.05; β = 0.09, *p* < 0.05) (Table 5). When controlling C-TONI-3, the manual dexterity and aiming and catching show a significant predictive effect on their digits RAN (β = 0.09, *p* < 0.05; β = 0.09, *p* < 0.05) (Table 6), color RAN (β = −0.14, *p* < 0.001; β = −0.13, *p* < 0.001) (Table 7), and object RAN (β = −0.23, *p* < 0.001; β = −0.10, *p* < 0.001) (Table 8). The results showed that both C-PPVT-R and balance are not predictors for all RAN in the multiple linear regression.

The results in Tables 5–8, wherein the manual dexterity of the children, show a significant predictive effect on their onset abilities of PA (β = 0.09, *p* < 0.001), digits RAN (β = −0.20, *p* < 0.001), color RAN (β = −0.14, *p* < 0.001), and object RAN (β = −0.23, *p* < 0.001). The results showed that when C-TONI and C-PPVT-R were considered, the manual dexterity score could both account for the variance in the onset subtest of PA and all RAN test scores. However, the scores of aiming and catching can only predict the RAN scores (see Tables 5–8).

### Co-morbidity analyses

Comorbidity of DCD with PA deficit. In total, 29.69% of the DCD children and 14.22% of the TD children were classified as having a PA deficit. The odds ratio was 2.55, meaning that the DCD children were 2.5 times more likely than the TD children to have a deficit in PA.

Comorbidity of DCD with RAN deficit. In total, 31.25% of the DCD children and 11.56% of the TD children were classified as having a RAN deficit. The odds ratio was 3.48, meaning that the DCD children were 3.5 times more likely than the TD children to have a deficit in RAN.

Comorbidity of PA deficit with RAN deficit. Among the TD children, 11% had a single PA deficit, 8% had a single RAN deficit, and 3% had a dual PA and RAN deficit; 78% had neither. Among the DCD children, 17% had a single PA deficit, 19% had a single RAN deficit, and 12% had a dual PA and RAN deficit; 52% had neither. There was a significant relationship between having a PA deficit and having a RAN deficit (χ^2^ = 19.1, *p* < 0.0001), consistent with the result of the regression analysis.

## Discussion

The present study examined the performances of the preschool DCD children and the TD children on PA and RAN. The notable findings can be summarized as follows. (1) The preschool DCD children presented poorer PA and slower naming than the TD children. (2) Both PA and RAN correlated with the children’s motor control abilities, especially that of manual dexterity. (3) Interestingly, PA did not correlate with motor control when RAN was controlled for. But, RAN continued to correlate significantly with motor control when PA was controlled for. (4) The DCD children were 2.5 times more likely than the TD children to have a deficit in PA. The DCD children were 3.5 times more likely than the TD children to have a deficit in RAN. (5) There was a significant relationship between having a PA deficit and having a RAN deficit (*r* = −0.27), and this was true for both the DCD and the TD children. Among the TD children, 11% had a single PA deficit, 8% had a single RAN deficit, and 3% had a dual PA and RAN deficit. Among the DCD children, 17% had a single PA deficit, 19% had a single RAN deficit, and 13% had a dual PA and RAN deficit.

Our findings show that the DCD children had poorer PA and RAN than the TD children, which are the same as the findings of Wylie and Bell (21). The authors observed that children with impaired motor skills performed poorly on measures of oral sentence reading and PA. Our findings also extended those of Cheng et al. (4), who found that preschool DCD children were three times more likely than TD children to have a developmental speech and language disorder. Because it is well known that both PA and RAN predict children’s reading development in later years, these findings call for special attention paid to the DCD children’s linguistic needs.

Wolf et al. proposed that the naming speed deficit was independent of the PA deficit and the former constituted the second deficit in developmental dyslexia (27). With respect to Chinese, RAN is a better predictor than PA of reading difficulties in Chinese-speaking children (28). We observed similar relationships of PA and RAN with DCD. First of all, we found a correlation between PA and RAN and this is consistent with the findings of previous studies (41, 42). In addition, we found that there remained a unique correlation between RAN and motor skills when PA was controlled for. But there was no unique correlation between PA and motor skills when RAN was controlled for. This pattern is similar to that observed in dyslexia alluded to earlier. It suggests that the skill to rapidly and automatically name words shares some processing components with the skill of motor control. This component is not phonological in nature. Moreover, the motor skill that is relevant in this relationship is manual dexterity, not aiming and catching, nor balancing. As a parallel, it is worth noting that Cheng et al. (4) found that manual dexterity, but not ball skills (i.e., aiming and catching) or balance, of MABC, was predictive of all scores on the speech and language tests.

Previous studies reported that developmental dyslexia in alphabetic languages was associated with sensorimotor deficits, balance, motor skills, time estimation, and cognitive deficit (43, 44). The dyslexic individuals in Chinese languages showed several impairments in the sensorimotor domain such as balance, motor skills, rapid processing, morphological awareness, and orthographic deficits (45). These findings indicated a universal cause of dyslexia across Chinese and alphabetic languages and showed abnormal activations in the different brain areas (45, 46). The sensorimotor impairments of both dyslexic individuals in Chinese languages and alphabetic languages are explained by the skill automatization deficit hypothesis (45). The authors proposed the Dyslexic Automatization Deficit hypothesis and attributed the deficits to an inability to become completely fluent in cognitive and motor skills. Our results are presented in multiple linear regression analysis, wherein the manual dexterity of the children shows a significant predictive effect on their onset abilities of PA, digits RAN, color RAN, and object RAN. However, the score of balance could only predict the onset of PA scores. Our finding is consistent with the Automatization Deficit hypothesis but in relation to manual dexterity. In similarities to our findings, studies have shown that the performance of fine motor skills in young children is predictive of reading success in older children (47). The core predictors of dyslexia in the preschool years included letter knowledge, PA, RAN, and fine motor skills (48). When the child goes into primary school, language skills become significant predictors, and motor skills can increase the prediction probability (48). However, manual dexterity seems more relevant to reading than balancing in our preschool children with probable DCD.

Neuroanatomically, the cortical areas responsible for the movement of the hand and the mouth region are in close proximity (49). Behaviorally, the subtest of manual dexterity consists of posting coins, threading beads, and drawing trail. It requires eye-hand coordination. Naming words, on the other hand, requires eye-mouth coordination. Both tasks involve visual perceptual-motor control. Fluent performances on them require the development of automaticity in such control. No matter, our findings and those of researchers, Cheng et al. (4) suggest that some processing component that underlies RAN, language control, and motor control may be that of automatic visual perceptual-motor coordination.

Currently, with cognitive neuroscience and meta-analysis research, it has been presented that children with DCD are correlated with complex series of neurological dysfunctions involving visual-spatial memory, eye-voice span, attention, reaction time, executive function, and other cognitive abilities (16, 17, 50–54). The studies found that the children with DCD manifest mainly in three principal components of executive function, namely, cognitive flexibility, inhibition control, and working memory. The automatic visual perceptual-motor coordination deviation in children with DCD shows significant differences in RAN task performance compared to PA. In the RAN task, this study found that, compared with the TD group, children with DCD attained significantly lower digit RAN, color RAN, and object RAN. The RAN is an automatic visual perceptual connection task that drives the search for the name and the speed of repeated responses, oculomotor, and oral-motor (50). It is the parallel circuitry that reflects both linguistic processes and executive aspects of cognition. Previous research suggests that RAN taps both visual-verbal (language domain) and processing speed (executive domain) contributions to reading (55). The early visual perceptual-motor deviations in preschool children with DCD in our study, mainly on deficits in the RAN and the PA are correlated with poor executive function and worse linguistic processes.

Phonological awareness (PA) and RAN ability are strong predictors of reading ability across groups of different ages, languages, and abilities (51). In the study, the preschool children with probable DCD performed worse in the IQ test (C-TONI-3 and C-PPVT-R), had poorer PA, and slower naming than the TD children. The comorbidity rate of the PA deficit and probable DCD was 29.7%, while the rate for the RAN deficit and probable DCD was 31.3%. The odds ratios of PA or RAN deficits were about 3-fold higher among the children with probable DCD compared to the TD children. This indicates that the comorbidity of motor, PA, and RAN deficits in preschool children with probable DCD are less able to recruit adequate executive functions and linguistic processes. It is a significant condition that requires attention from clinicians and teachers. Therefore, a comprehensive examination of the motor and cognitive function of preschool children is important. Such examination may assist in the identification of children with cognitive and motor difficulties and allows early intervention. Further study is suggested to follow up on the outcomes of academic learning and social adaptation in children with probable DCD with or without deficits in PA/RAN identified at preschool age. It is suggested that intervention for preschool children with probable DCD warrants both the visual-verbal (language domain) and processing speed (executive domain) approaches.

The limitations of our study include a limited scope of assessment and limited resources of participants. The motor assessment in the study included only MABC-2, which contains only eight test items. Studies have noted that different motor test batteries may identify children with different motor impairments (15, 35). Further study may include the Bruininks-Oseretsky Test of Motor Proficiency (BOTMP) for a more comprehensive motor test (56). The participants were recruited from preschools in only one municipal city in Taiwan. Therefore, such limitations may affect the generalization of our study. This issue will be addressed in future studies.

## Conclusion

Coming back to the issue of deficit, our results showed that 22% of the TD children, but 48% of the DCD children, had a single or dual PA/RAN deficit. The high rate of PA/RAN deficit would put the preschool DCD children at a much higher risk of having reading difficulty when they enter school. Indeed, Cheng et al. (4) reported that preschool DCD children were three times more likely than TD children to have a developmental speech and language disorder. This early visual perceptual-motor profile of DCD children has not been well recognized before. Early intervention is called for to address the special linguistic needs of these children.

## Data availability statement

The original contributions presented in this study are included in the article/supplementary material, further inquiries can be directed to the corresponding author.

## Ethics statement

The studies involving human participants were reviewed and approved by Institutional Review Board of Kuang Tien General Hospital. Written informed consent to participate in this study was provided by the participants’ legal guardian/next of kin.

## Author contributions

H-CC designed the study, collected the data, conducted most of the analyses, provided training for the experimenters, and wrote the first draft of the manuscript. R-JC codesigned the study and contributed to the writing and revising of the first draft. P-YY helped in the analyses of the results and edited the language. All authors contributed to the article and approved the submitted version.
